# Breathable and Flexible Piezoelectric ZnO@PVDF Fibrous Nanogenerator for Wearable Applications

**DOI:** 10.3390/polym10070745

**Published:** 2018-07-05

**Authors:** Minji Kim, Yuen Shing Wu, Edwin C. Kan, Jintu Fan

**Affiliations:** 1 Department of Fiber Science and Apparel Design, Cornell University, Ithaca, NY 14853, USA; mk988@cornell.edu (M.K.); yw526@cornell.edu (Y.S.W.); 2 School of Electrical and Computer Engineering, Cornell University, Ithaca, NY 14853, USA; kan@ece.cornell.edu

**Keywords:** nanogenerator, piezoelectric, wearable, PVDF, fiber, electrospin, ZnO, nano, hydrothermal growth, breathable

## Abstract

A novel breathable piezoelectric membrane has been developed by growing zinc oxide (ZnO) nanorods on the surface of electrospun poly(vinylidene fluoride) (PVDF) nanofibers using a low-temperature hydrothermal method. Significant improvement in the piezoelectric response of the PVDF membrane was achieved without compromising breathability and flexibility. PVDF is one of the most frequently used piezoelectric polymers due to its high durability and reasonable piezoelectric coefficient values. However, further enhancement of its piezoelectric response is highly desirable for sensor and energy-harvester applications. Previous studies have demonstrated that piezoelectric ceramic and polymer composites can have remarkable piezoelectric properties and flexibility. However, devices made of such composites lack breathability and some present health risks in wearable applications for containing heavy metals. Unlike other piezoelectric ceramics, ZnO is non-toxic material and has been widely used in many applications including cosmetics. The fabrication of ZnO@PVDF porous electrospun membrane involves a simple low-temperature ZnO growth in aqueous solution, which does not weaken the polarization of PVDF created during electrospinning in the high electric field.

## 1. Introduction

Interest in wearable piezoelectric materials has grown tremendously due to the increasing need for powering mobile devices, achieving sustainable operations, and measuring long-term biometric data [1,2,3]. Piezoelectric polymers are attractive for wearables due to their flexibility and conformability over piezoelectric ceramic materials [4,5,6,7]. Poly(vinylidene fluoride) (PVDF) and its copolymers are known to have very large and stable ferroelectric, piezoelectric, and pyroelectric properties among polymers. Also, it has high resistivity to daily chemicals which makes it safe to use against cleaning agents, skin products, and sweat [8]. However, PVDF and its copolymers still have significantly lower piezoelectric coefficients than piezoelectric ceramics [9,10,11,12], and further improvement is desirable for sensor and energy-harvester applications. Many researchers have demonstrated that incorporating piezoelectric ceramics into PVDF to create a composite material can achieve higher piezoelectric constant and excellent electromechanical coupling factor [13,14,15,16,17,18,19].

Chemical resistivity of PVDF also brings forth stability and safety for use next to human skin. To select piezoelectric ceramics to be embedded in PVDF matrix, the potential health hazard is one of the most important factors to be considered [20,21]. Lead zirconate titanate (PZT) is one of the most common piezoelectric ceramics due to its high piezoelectric response. However, PZT poses strong risks for containing highly toxic lead. Lead-free piezoelectric materials have been extensively researched to replace PZT [22,23,24,25], where zinc oxide (ZnO) shows strong potential for next-to-skin applications. ZnO is widely used in sunscreens and its application is approved by FDA [26,27,28,29]. Also, ZnO is one of the ceramics with lowest hydrothermal reaction temperatures among lead-free piezoelectric ceramics [30,31,32,33]. The low reaction temperature was preferred as it allows the avoidance of an additional poling step after the addition of ceramics to the polymer matrix. To overcome the brittleness and limited yield strength of inorganic materials, ZnO has been added to non-porous polymer matrix as thin films to fabricate more flexible nanogenerators [34,35,36,37]. These nanogenerators have reasonable piezoelectric performance, but the films are not breathable, which limits their use in wearable applications.

Piezoelectric polymers can be made into thin films or fibers. Fibrous materials are both breathable and flexible, and therefore more suitable for wearable applications than thin films. Also, one-dimensional PVDF fibers can have higher piezoelectric energy conversion efficiency than films [38,39]. However, so far piezoelectric fibrous materials have been assembled with at least two layers of non-breathable materials. For example, solid metal films were used as electrodes, and plastics as an external casing to protect from ambient contamination and to ensure good electrical contact. These material additions compromise the flexibility and breathability of the fibrous material required for wearable applications [15,16,17,18].

Here we present a piezoelectric nanofibrous material with improved piezoelectric responses by growing ZnO nanorods onto the PVDF fiber surface (ZnO@PVDF) through a facile hydrothermal process. We have also fabricated a truly breathable and flexible fibrous nanogenerator with conductive textile electrodes without impermeable casing.

## 2. Materials and Methods

### 2.1. Preparation of PVDF Nanofiber Mats

PVDF nanofiber mats were fabricated by electrospinning. The polymer solution of 20 wt % of PVDF (*M*_w_ = 180,000, Millipore Sigma, Burlington, MA, USA) was prepared with *N*,*N*-dimethylformamide (DMF, Macron Fine Chemicals, Center Valley, PA, USA) and acetone (Fisher Chemical, Waltham, MA, USA). The PVDF solution was prepared by adding PVDF pellets to the DMF-acetone solvent mixture (7/3 *v*/*v*), and then stirred with a magnetic stir bar on a hotplate at 70 °C, 150 rpm for three hours. The PVDF solution was withdrawn into a syringe with spinneret needle gauge size 23 and was then pushed by a syringe pump (SK-500 III, Shenzhen Shenke Medical, Nanshan, China) with a rate of 1.0 mL/h. A high voltage of 14 kV was applied by a DC power supply (Matsusada Precision, Kusatsu-shi, Japan) to the needle. Fibers were accumulated on a grounded 100-mm-diameter collector rotating at 200 rpm. The distance between the cylindrical collector and spinneret was 10 cm. A Teflon tube was used to connect the syringe and the needle, and the needle was moved laterally with a stroke of 18 cm and approximately four strokes per minute to obtain wide and uniform nanofiber mats.

### 2.2. Hydrothermal Growth of ZnO Nanorods

Our ZnO nanorod growth method was developed based on the previous studies [30,40,41,42]. Electrospun PVDF fibers are at least one order of magnitude smaller than cotton or nylon microfibers used in the previous studies. Therefore, our seed and growth solution were further diluted than those reported methods so that the resulting ZnO nanorods had smaller diameters than the fiber. PVDF is also chemically more inert than cellulose or nylon, with more difficult seeding processes and fewer nucleation sites. During the seeding step of ZnO deposition, it is critical to form hexagonal nanorods arranged vertically to the substrate surface for maximum strain rate transfer [40]. Therefore, the seeding process was repeated three times to provide sufficient nucleation sites. The oven temperature for seed curing and growth process was lowered to 60 °C to inhibit heat relaxation of PVDF and to preserve the piezoelectric polarization of the electrospun fibers, thus avoiding the additional poling process [43]. Equimolar of hexamethylenetetramine and zinc nitrate hexahydrate in the growth solution was replaced by a higher concentration of hexamethylenetetramine to yield the preferred ratio of the polar and non-polar surface of ZnO crystal for higher piezoelectric responses [41,44].

#### 2.2.1. Preparation of ZnO Seed Solution

Zinc acetate dihydrate (7.5 mmol, 1.6462 g) was dissolved in 150.0 mL of isopropyl alcohol, and the solution was stirred vigorously at 85 °C for 15 min. Then, trimethylamine (7.5 mmol, 0.7637 g) was added dropwise to the solution which subsequently became clear. The solution was then stirred again at 85 °C for 10 min and incubated at room temperature without stirring for three hours. Isopropyl alcohol (600 mL) was added to dilute the solution to 10 mM before use.

#### 2.2.2. Preparation of ZnO Growth Solution

Hexamethylenetetramine (HMT, 66.7 mmol, 9.3457 g) was dissolved in 400 mL of deionized (DI) water, and the solution was stirred for 10 min. Zinc nitrate hexahydrate (ZNT, 40.0 mmol, 11.8991 g) was added to this solution, which was followed by stirring for 24 h. All processes were performed at room temperature. DI water was added to 10-fold dilution before use (16.7 mM of HMT, 10 mM of ZNT).

#### 2.2.3. Growth of ZnO Nanorods

PVDF nanofiber membrane was placed in between two Teflon frames (45 mm × 105 mm inner dimension, 15 mm wide, 3 mm thick) for structural stability. The seeding procedure was done in three rounds comprising of immersing in seed solution and curing in 60 °C oven. For the first round of seeding, the membrane was immersed into the seed solution (10 mM) for 20 min, then cured in 60 °C oven for 20 min. For second and third rounds, immersion was for two minutes, and curing was the same 20 min. After the seeding process, the PVDF nanofibers in the Teflon frame was dipped into 400 mL of growth solution (16.7 mM of HMT, 10 mM of ZNT), and placed in 60 °C oven for 6 h. The sample stayed in the inert oven for gradual cooling down to the room temperature, which typically lasted for 11–13 h. The samples were then rinsed with running DI water for five minutes and dried in air.

### 2.3. Piezoelectric Response Measurements

The piezoelectric testing module was assembled by sandwiching the piezoelectric nanofiber mat between two electrodes made by either conductive fabric (cotton and silver blend double jersey knit purchased from LessEMF, Latham, NY, USA) or aluminum foil.

Piezoelectric performance of the electrospun samples was evaluated by periodic tensile testing in a customized setup with a motorized actuator controlled by a controller and a module (ni-cRIO 9036 and ni-9503, National Instruments, Austin, TX, USA), a pressure sensor (LC201-300/N, Newport Electronics, Santa Ana, CA, USA), electrometer (6517B, Keithley Instruments, Cleveland, OH, USA), multimeter (34470A, Keysight, Santa Rosa, CA, USA) and programmable DC power supply (9130, BK Precision, Yorba Linda, CA, USA). The testing head movement was set to 1 Hz, and impact pressure was set to 0.10 MPa which is within the human foot pressure range of 0–0.20 MPa [45,46,47], although the applications are not limited to shoes. LabView (National Instruments, Austin, TX, USA) was used to control all components and to record voltage and current outputs in a synchronous manner.

### 2.4. Characterization Methods

Field-emission scanning electron microscope (FESEM) images were taken using LEO 1550 (ZEISS, Oberkochen, Germany) with an accelerating voltage of 3 keV. To extract the detailed geometry with better focus and to reduce the charging effect under FESEM, the samples were partially sputter coated with palladium/gold. The average PVDF fiber diameter and ZnO nanorod length and diameter were determined by FESEM images over 30 fibers with the ImageJ software, NIH, Bethesda, Rockville, MD, USA [48].

Crystallography of PVDF nanofibers and ZnO nanorods was examined by X-ray diffraction (XRD) with the powder diffractometer (D8 GADDS, Bruker), where Cu-*K*α radiation (λ = 1.54 Å) at a 0.02° scanning step and an operating voltage of 40 kV and current of 40 mA are used.

Fourier-transform infrared (FTIR) spectroscopy from 4000 to 650 cm^−1^ (Frontier FTIR, PerkinElmer, Waltham, MA, USA) was performed at room temperature to evaluate the polymer crystalline phase. FTIR spectra were collected with 16 scans and a resolution of 4 cm^−1^.

Thermal gravimetric analyses (TGA) were used to determine the amount of ZnO added to PVDF nanofibers (Q500 Thermogravimetric Analyzer, TA Instruments, New Castle, DE, USA) by heat removal of PVDF. Samples were heated up from room temperature to 990 °C with the rate of 10 °C/min on a ceramic pan under a nitrogen ambient.

The water vapor permeability (WVP) was measured with an upright cup method according to BS 7209 [49,50,51]. The tests were conducted in a conditioned room with 22.5 ± 0.5 °C temperature and 59.0 ± 1.5% relative humidity. Samples were conditioned in the conditioning room over 24 h. The turntable holding cups were rotated uniformly to avoid the formation of still air layers above the test dishes at 2.3 m/min. Aluminum cups used in this experiment had an inner diameter of 83 mm, an outer diameter of 90 mm, inner cup thickness of 18.5 mm, and outer cup thickness of 20 mm. Each cup contained 18 g of DI water and a triangular support was used to prevent samples sagging into the cup. Samples were tested over 60 h to determine the water mass loss over the time as the water vapor permeability of test samples.

## 3. Results

PVDF has a high piezoelectric coefficient among piezoelectric polymers and is a semi-crystalline polymer with up to five different crystal phases (α, β, γ, δ, and ε) [8]. Two most common phases are the non-polar α-phase (TGTG, trans-gauche), and the polar β-phase (TTTT, all-trans) [52,53]. Among all phases, the β-phase has the most favorable piezoelectric property with the highest dipolar moment per unit cell [17,52]. There have been many studies seeking to obtain a higher percentage of the β-phase in PVDF. In terms of in-situ poling during fiber formation, drawing with an electric field during the melt spinning [53] and various types of electrospinning [17,54,55,56,57] have shown to produce fibers with a high β-phase percentage. With electrospinning, the post-processing poling step is not necessary due to the high field within the process. The resulting nanofiber mats also have large surface area due to small fiber diameters [58].

### 3.1. ZnO Growth on PVDF Nanofibers

**Figure 1:**
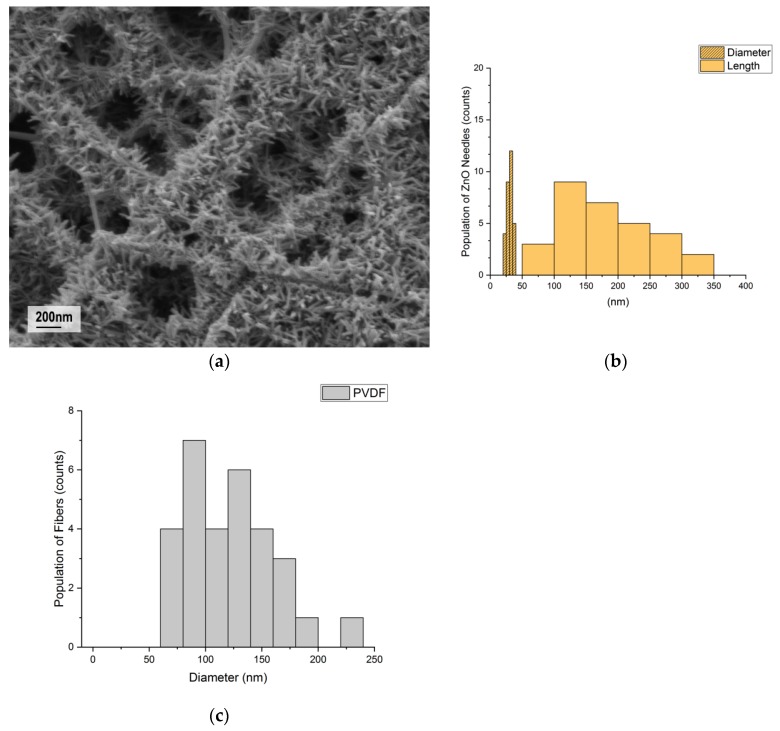
(**a**) An FESEM image of the ZnO nanorods grown on the surface of the electrospun PVDF nanofiber; (**b**) histogram of the length and diameter distributions of ZnO nanorods; (**c**) histogram of the diameter distribution of electrospun PVDF nanofibers.

The nanostructure of PVDF nanofibers and ZnO nanorods were first examined under FESEM. As described in the experimental section, the ZnO nanorods were grown on the PVDF fiber surface by the hydrothermal growth method. The FESEM image in Figure 1a shows the morphology and the size distribution of the electrospun PVDF nanofibers and ZnO nanorods. Figure 1a shows the area of successful ZnO nanorods growth. ZnO nanorods had an average length of 183 ± 153 nm and diameter of 30 ± 9 nm (Figure 1b), and the electrospun PVDF nanofibers with random fiber alignment had an average diameter of 120 ± 100 nm (Figure 1c).

**Figure 2:**
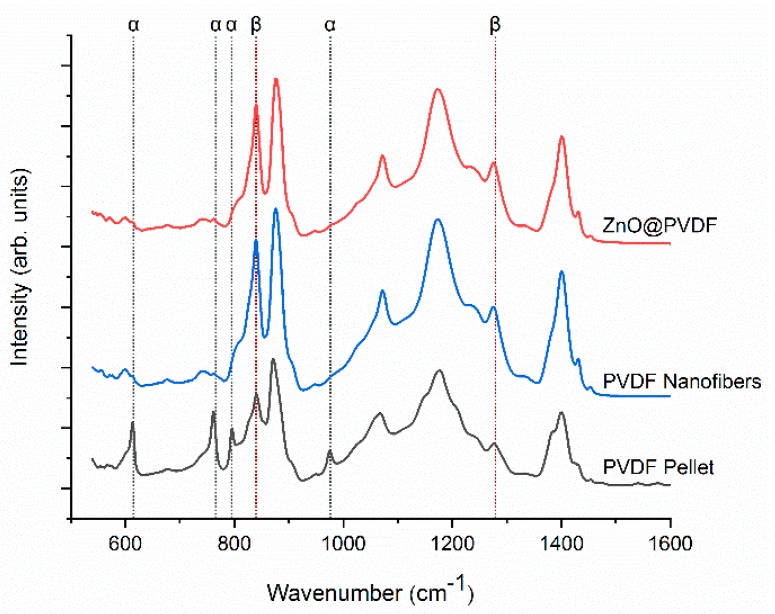
FTIR spectra showing the effect of electrospinning and hydrothermal growth on PVDF crystalline phases.

FTIR spectra were studied to analyze the PVDF crystalline phases under the electrospinning and hydrothermal growth process. FTIR spectra of the PVDF polymer pellets, electrospun PVDF nanofibers, and ZnO@PVDF nanofibers are compared in Figure 2. The pellet exhibits strong peaks corresponding to non-polar phase α crystalline at 615 cm^−1^ (α, CF_2_ bending and skeletal bending), 766 cm^−1^ (α, CF_2_ bending), 795 cm^−1^ (α, CF_2_ rocking), 855 cm^−1^ (α, CF_2_ out-of-plane deformation), and 976 cm^−1^ (α, CH out-of-plane deformation) [59,60]. After electrospinning, peaks at 840 cm^−1^ (β, CH_2_ rocking) and 1279 cm^−1^ (β, CF out-of-plane deformation) got stronger, and peaks corresponding to α crystalline got weaker. Hydrothermal growth of ZnO nanorods did not depolarize PVDF fibers, and similar FTIR spectra of nanofibers confirmed this before and after the growth process. The maintained intensity of β crystalline peaks indicates that PVDF polarization does not relax during hydrothermal growth. Furthermore, the β-phase percentage can be quantified using the following equation:

(1) $$\;F_{\beta }\;=\;\frac{X_{\beta }\;}{X_{\alpha }\;+\;X_{\beta }}\;=\;\frac{A_{\beta }}{\left(K_{\beta }/K_{\alpha }\right)A_{\alpha }\;+\;A_{\beta }}$$

where $F_{\beta }$ represents the PVDF β-phase percentage, $A_{\alpha }$ and $A_{\beta }$ are their absorption bands at 766 and 840 cm^−1^, and $K_{\alpha }$ and $K_{\beta }$ are the absorption coefficients at the respective wavenumber, which are $6.1\times 10^{4}$ and $7.7\times 10^{4}$ cm^2^·mol^−1^, respectively [52,60,61]. The calculated β-phase percentage for PVDF pellets was 49.4%, for the electrospun PVDF nanofibers was 83.8%, and for the ZnO@PVDF nanofibers was 80.2%. The hydrothermal growth temperature of ZnO nanorods on PVDF fibers was set to 60 °C, because at higher temperatures PVDF polymer tends to relax, reducing its piezoelectric responses. The Curie temperature—when the piezoelectric response of PVDF polymers vanishes—is about 165–170 °C [43]. From the comparison of the three FTIR spectra, it can be concluded that electrospinning process successfully produced PVDF fibers with a high percentage of β phase and ZnO nanorod growth did not depolarize the PVDF nanofibers.

**Figure 3:**
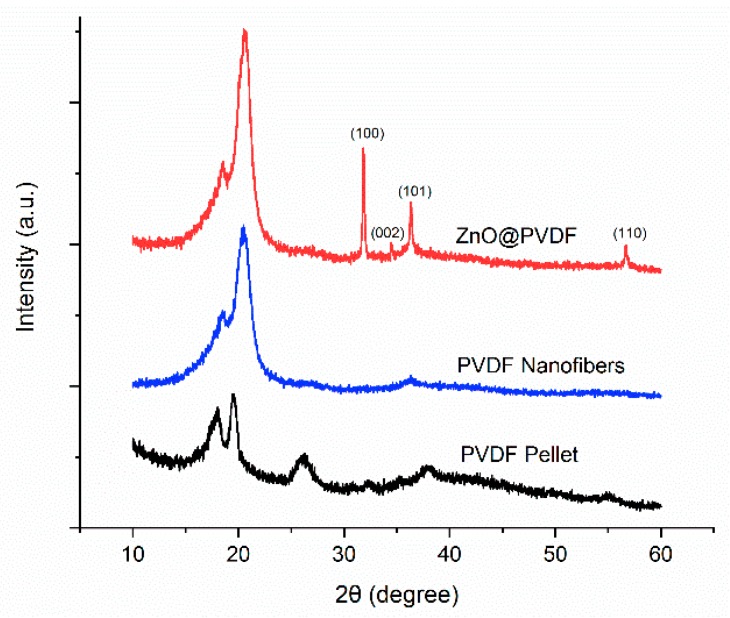
XRD spectra of the PVDF pellet, PVDF membrane, and ZnO@PVDF.

Figure 3 shows the X-ray diffraction patterns of the PVDF pellet, electrospun PVDF nanofibers, and ZnO@PVDF composite nanofibers. Electrospun PVDF nanofibers exhibited strong peak 2θ at 20.4° which corresponds to the β phase crystalline. ZnO@PVDF composite nanofibers show four reflection peaks at 2θ values of 31.9° (100), 34.5° (002), 36.3° (101), and 56.7° (110) which can be indexed as the hexagonal wurtzite structure [62,63]. The wurtzite structure is the most thermodynamically stable form of anisotropic hydrothermal growth owing to the presence of polar and nonpolar surfaces, with a natural tendency to minimize polar surface and surface energy [41,64]. No diffraction peaks from any other impurity phases are found, confirming that only single-phase hexagonal ZnO is present. Peaks corresponding to the (100), (002), (101), and (110) planes of ZnO@PVDF composite fibers confirmed the hydrothermal growth of ZnO wurtzite crystals at 60 °C [65,66]. No notable difference of PVDF α, β crystalline peak before and after hydrothermal ZnO growth was observed. Therefore, our hydrothermal growth in the relatively low temperature resulted in the successful growth of ZnO without depolarizing the electrospun piezoelectric nanofibers.

**Figure 4:**
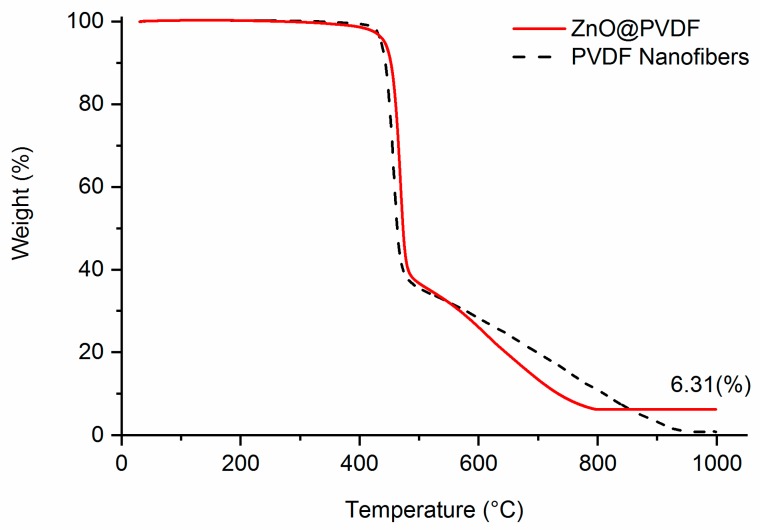
TGA of PVDF membrane, and ZnO@PVDF.

The amount of ZnO added onto PVDF fibers was evaluated by TGA (Figure 4). It was calculated by the weight loss of the samples at the end of the heating cycle. The composite sample contained about 6.31 wt % of ZnO. The ZnO weight percentage in the composite is relatively small because ZnO nanorod growth is limited to the surface of the nanofiber membrane.

### 3.2. Piezoelectric Measurements

**Figure 5:**
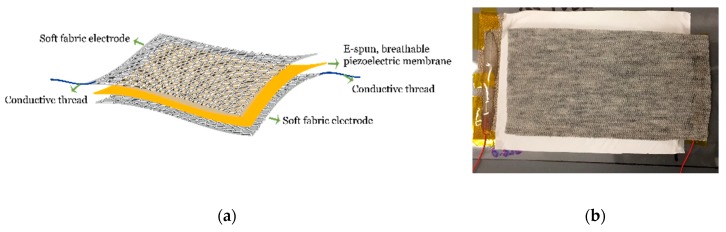
The breathable fibrous nanogenerator (**a**) schematic illustration and (**b**) photo.

Figure 5a shows a schematic illustration of the breathable fibrous nanogenerator which comprises a piezoelectric nanofiber membrane sandwiched by two conductive knitted fabric electrodes. Wires were connected to the electrodes using conductive ink and epoxy. A photo of the device is shown in Figure 5b. The average thicknesses of the nanofiber membrane, the aluminum foil and the conductive knitted fabric electrode were 59 ± 14.5, 24.2 ± 5.8, and 599 ± 14.3 μm, respectively. The sheet resistances of the conductive knitted fabric and the aluminum foil were 1459 ± 6 and 413 Ω, respectively.

#### 3.2.1. Comparison of Aluminum Foil and Conductive Knitted Fabric as Electrodes

**Figure 6:**
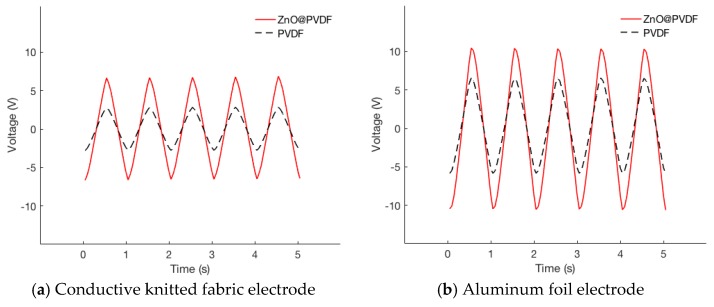
Open-circuit voltage measurements under 0.10 MPa impact of 1 Hz frequency: (**a**) conductive knitted fabric electrode nanogenerator; (**b**) aluminum foil electrode nanogenerator. The black lines are from PVDF, while the red lines from ZnO@PVDF.

Aluminum foil and conductive knitted fabric were used as electrodes for comparison. Four types of nanogenerators were made with two types of piezoelectric nanofibrous membranes, PVDF and ZnO@PVDF, and two types of electrodes, that is to say, aluminum foil and conductive knitted fabric. Open-circuit voltages were measured to evaluate the two electrode materials. Figure 6 shows the cyclic responses of the piezoelectric nanogenerators when the module is subjected to repeated compressive impact. The average values, standard deviation, and coefficient of variation (CV) of the maximum open-circuit voltage when the piezoelectric nanogenerators were subjected to repeated compressive impact are listed in Table 1. A custom-made cyclic compression tester was used to impact the nanogenerator with an effective area of 15 cm^2^, a frequency of 1 Hz and pressure of 0.10 MPa, emulating foot pressure during walking.

Open-circuit voltages for aluminum foil electrodes were found to be higher than those for conductive knitted fabric electrodes for both PVDF and ZnO@PVDF. Also, the variation of measurements was larger for aluminum foil than for the conductive knitted fabric. A possible explanation for the conductive fabric electrodes giving more stable and reproducible results is that it provided better adherence between the piezoelectric membrane and the electrodes. We opted not to use any additional casing to press adherence of electrodes and piezoelectric membrane layers to maintain the best flexibility and breathability, and thus the air gap between the layers can exist due to the lack of external forces. The flexibility and stretch-ability of the materials determine the amount of air gap created. Such properties can be measured with the Fabric Assurance by Simple Testing (FAST) system [67]. The bending and shear rigidity of the conductive knitted fabric were 0.87 ± 0.05 μN∙m and 21.42 N/m, respectively. For aluminum foil, the values were beyond the measurement range of the FAST system. The higher absolute measurements with aluminum foil electrodes are due to the aluminum sheet being more conductive than porous fabric. The open-circuit voltage of the nanogenerators in our work, which is less affected by the air gap existence than the load current, was significantly higher than those reported in the literature [15,16,17].

#### 3.2.2. Improvement of Piezoelectric Property after Growing ZnO Nanorods on PVDF Fibers

Since conductive knit electrodes provided more consistent piezoelectric responses than aluminum foil, conductive knits were used to construct breathable nanogenerator made of as-spun PVDF and ZnO@PVDF for further evaluation.

**Figure 7:**
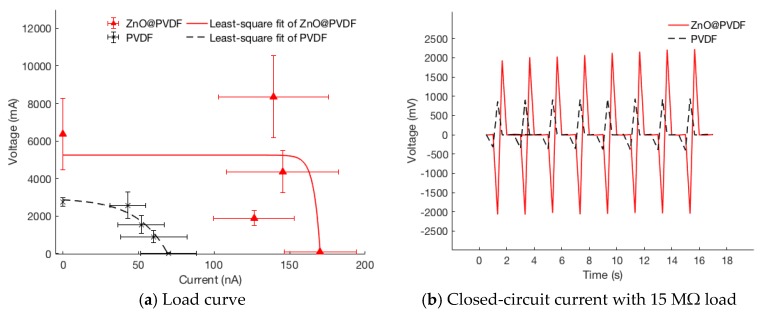
Nanogenerator characteristics for PVDF and ZnO@PVDF 15 cm^2^ fiber mat: (**a**) load curves with resistive loads of 0.47, 15, 30, and 60 MΩ; (**b**) transient closed-circuit current measurements with the 15 MΩ resistive load.

Closed-circuit currents of the nanogenerators with different resistive loads were compared, and the closed-circuit voltages and the power density were derived accordingly. Table 2 listed the average values of the maximum peak currents with resistive loads of 0.47, 15, 30, and 60 MΩ. Figure 7a shows the nanogenerator load curve with all data points from different loads. Nonlinear least-square fitting was performed on the following equation:

(2) $$V\;=\;V_{\mathrm{oc}}\left(1-\exp\left(\left(I-I_{\mathrm{sc}}\right)/I_{0}\right)\right)$$

where V represents the measured voltage, I the measured current, and $V_{\mathrm{oc}}$, $I_{\mathrm{sc}}$, and $I_{0}$ are the extracted parameters of the open-circuit voltage, short-circuit current, and *I-V* sharpness fitting. Figure 7b shows a typical transient current responses when the sample was subjected to cyclic compressive impact with a resistive load of 15 MΩ.

As can been seen from Table 1 and Table 2 as well as Figure 6 and Figure 7, ZnO@PVDF nanogenerators produced significantly greater power output than PVDF nanogenerators under all load conditions. Growing ZnO nanorods on the surface of electrospun PVDF nanofibers have increased open-circuit voltage by 231%, and load currents by 244% and 210% for 470 kΩ and 15 MΩ, respectively.

### 3.3. Material Breathability

The breathability of piezoelectric fibrous nanogenerators, as well as that of the constituent materials, were evaluated by measuring the water vapor permeability for the nanogenerators, which consisted of an electrospun PVDF or ZnO@PVDF membrane sandwiched by two layers of conductive knitted fabrics. An upright cup method is employed in accordance with the BS 7209 standard. A cotton woven fabric was also tested at the same time for a benchmark. The water vapor permeability (WVP) was calculated using the following formula:

(3) $$\mathrm{WVP}\;=\;\frac{24\;M}{At}$$

where WVP is the rate of water vapor permeability (g·m^−2^·day^−1^), *M* the loss in mass (g), *t* the time duration (h), and *A* the area of the exposed test fabric which is equal to the internal area of the dish (m^2^). The results were averaged from three specimens over 60 h.

From Table 3, all materials used in the nanogenerator had similar water vapor permeability, which is also comparable to that of the cotton woven fabric. Electrospun PVDF membrane’s water vapor permeability decreased slightly after ZnO growth on the surface, but the difference was small. Nanofibrous materials showing smaller WVP than microscale fabrics aligns with other works related to theory and experiment of gas diffusion behavior of nanoscale and microscale fibrous media [68,69]. The conductive knitted fabric was more breathable than the electrospun membranes or woven cotton. The three-layer nanogenerator assembly by PVDF had the lowest WVP. However, it is interesting to see that the resulting WVP was not reduced proportionally to the stack of the individual layers, but only slightly less than each layer. That the nanogenerator assembly WVP was not proportional to the number of layers may be due to the fact that the conductive knitted fabric had much larger pores and the still air layer associated with the of nanogenerator assembly is not much different from that on the single layer.

## 4. Discussion

High piezoelectric response, breathability, and flexibility are essential to nanogenerators for wearable applications. The present study demonstrated that breathability and flexibility of the fibrous nanogenerators can be achieved by assembling a layer of an electrospun piezoelectric nanofibrous membrane with two layers of conductive knitted fabric and without the use of rigid plastic casing. It further showed that the piezoelectric responses of PVDF fibrous nanogenerators can be significantly enhanced (up to 2.3 times regarding open-circuit voltage) by growing ZnO nanorod onto the PVDF fibers via a low-temperature hydrothermal process which does not weaken the polarization of PVDF created during electrospinning.

Our nanogenerator performance was compared with the previous methods in Table 4. While the open circuit voltage of the breathable ZnO@PVDF fibrous nanogenerators exceeded the reported non-breathable fibrous nanogenerators with rigid plastic casing, the power generated was relatively lower due to the lower load current, probably due to imperfect contact between the fabric electrodes and the piezoelectric membrane. Having an external plastic casing can ensure good contact of electrodes and piezoelectric fibers by applying constant stress, but it makes the nanogenerator unbreathable and inflexible for wearable applications. Assembling the fibrous nanogenerators without using an impermeable and less flexible plastic casing while maintaining excellent contacts between the layers remains a challenge which should be further investigated for wearable applications of next-to-skin nanogenerators.

## Figures and Tables

**Table 1:** Open-circuit voltages for piezoelectric samples under 0.10 MPa impact of 1 Hz frequency.

Materials	Conductive knit electrode			Aluminum foil electrode		
	Average (V)	Standard deviation σ (V)	Coefficient of variation	Average (V)	Standard deviation σ (V)	Coefficient of variation
PVDF	2.76	0.22	0.08	6.26	3.51	0.56
ZnO @PVDF	6.36	1.89	0.30	10.84	6.12	0.56

**Table 2:** Measured closed-circuit current and voltage averages with conductive knitted fabric electrodes.

		0.47 MΩ	15 MΩ	30 MΩ	60 MΩ
PVDF	Current (nA)	69.78	60.06	51.79	42.77
	Power Density (nW/cm^2^)	0.15	3.61	5.36	7.32
	Voltage (mV)	32.80	900.96	1553.63	2566.49
	Standard Deviation σ (mV)	8.59	328.30	460.01	704.94
	Coefficient of Variation	0.26	0.36	0.30	0.27
ZnO@PVDF	Current (nA)	170.10	126.33	145.30	139.36
	Power Density (nW/cm^2^)	0.91	15.96	42.22	77.69
	Voltage (mV)	79.95	1894.88	4359.03	8361.61
	Standard Deviation σ (mV)	11.20	399.01	1116.67	2176.22
	Coefficient of Variation	0.14	0.21	0.26	0.026

**Table 3:** Water vapor permeability (WVP) for various membranes and fabrics.

WVP (g·m^−2^·day^−1^)	PVDF	ZnO@PVDF	Conductive knit	Nanogenerator assembly by PVDF	Cotton
Average	629.00	623.68	645.57	608.20	611.82
Standard Deviation σ	31.95	16.92	26.98	15.05	32.02
Coefficient of Variation	0.05	0.03	0.04	0.03	0.05

**Table 4:** Performance comparison of ZnO@PVDF with conductive knitted fabric electrodes to other works of flexible and breathable films.

	(units)	ZnO@PVDF	Lee et al. [16]	Zeng et al. [15]	Fang et al. [32]
Material		PVDF, ZnO	PVDF, ZnO	PVDF, NaNbO_3_	PVDF
Fabrication method		electrospinning	dip coating	electrospinning	needleless electrospinning
Ceramic addition		growth	growth	filler	
Pressure or strain	(MPa or %)	0.1 MPa	<1%	0.2 MPa	0.05 MPa
Frequency	(Hz)	1	~1	1	5
Open-circuit voltage	(V)	6.4	0.1	3.2	2.6
Closed-circuit current	(μA)	0.17	-	4.2	4.5
Current density	(nA/cm^2^)	11.34	10	672	2250
Current resistive load	(kΩ)	470	-	470	-
